# Identification of Peripheral Anterior Synechia by Corneal Deformation Using Air-Puff Dynamic Anterior Segment Optical Coherence Tomography

**DOI:** 10.3389/fbioe.2022.856531

**Published:** 2022-04-01

**Authors:** Shuling Ye, Chenhong Bao, Yulei Chen, Meixiao Shen, Fan Lu, Shaodan Zhang, Dexi Zhu

**Affiliations:** ^1^ School of Ophthalmology and Optometry, Wenzhou Medical University, Wenzhou, China; ^2^ Linhai First People’s Hospital, Taizhou, China

**Keywords:** optical coherence tomography, air puff, peripheral anterior synechia, angle closure glaucoma, imaging

## Abstract

Indentation gonioscopy is commonly used in the clinic to evaluate peripheral anterior synechia (PAS) of angle closure glaucoma (ACG). The examination requires contacting with the cornea, resulting in an uncomfortable feeling for patients, and it only provides qualitative outcomes which may be affected by subjective judgment of the clinicians. Previous studies had reported to identify the presence of PAS by measuring the changes of morphological parameters of the anterior chamber angle (ACA) under the pupillary light reflex, by anterior segment optical coherence tomography (AS-OCT). However, this method was invalid for some subjects who had low sensitiveness to light. This article describes an air-puff dynamic anterior segment optical coherence tomography (DAS-OCT) system that can evaluate the presence of PAS in a non-contact approach. The peripheral cornea is deformed by an air puff jetted from the DAS-OCT, causing a transfer of force to the ACA, just as how indentation gonioscopy works. The dynamic changes of the ACA before and after the air puff are recorded by OCT. Ten eyes of normal subjects were enrolled in this study to validate the repeatability and availability of the measurements. Then, ten samples of the ACA from five subjects with ACG were recruited and were assigned into two groups, the non PAS group (NPAS) and PAS group, according to the results of gonioscopy. The ACA structural parameters including the angle opening distance at 750 μm to the scleral spur (AOD750) and the trabecular-iris space area at 750 μm anterior to the scleral spur (TISA750) were then calculated automatically by a custom-written algorithm. The intraclass correlation coefficient (ICC) of measured parameters was all above 0.85 for normal subjects, exhibiting good repeatability. For patients, both parameters showed significant differences between the two groups after the air puff, while no differences were observed before the air puff. AOD750dif and TISA750dif between two groups showed more significant differences, indicating that they could be used as indicators to identify the presence of PAS. In conclusion, the DAS-OCT system proposed in this study is demonstrated effective to identify the presence of PAS by measuring the changes of the ACA *via* a noncontact approach. It shows great potential for applications in guidance for diagnosis of angle closure glaucoma.

## Introduction

Primary angle closure glaucoma (PACG) is responsible for the vast majority of glaucoma blindness in China and has become a significant burden on health care systems and the society (Foster and Johnson, 2001; Tham et al., 2014; Song et al., 2017). The peripheral anterior synechia (PAS), which blocks the aqueous humor outflow pathway, was thought as one of the primary causes for PACG (Aung et al., 2005; Lee et al., 2006; Sun et al., 2017). Nowadays, indentation gonioscopy is commonly used in the clinic to evaluate the presence and extent of PAS in angle closure glaucoma (ACG). During the examination of indentation gonioscopy, the anterior chamber angle (ACA) open, and more structures in the ACA can be observed caused by cornea deformation for non-PAS (NPAS) patients. On the contrary, no more information will be observed by clinicians for the PAS patients. However, indentation gonioscopy is a contact examination on the cornea, resulting in an uncomfortable feeling for most patients. Besides, it only provides qualitative outcomes which may be affected by the experience and proficiency level of the clinicians (Foster et al., 2000; Phu et al., 2019).

Anterior segment optical coherence tomography (AS-OCT) has been reported to evaluate PAS by measuring the changes of morphological parameters of the ACA under the pupillary light reflex in previous studies (Leung et al., 2007; Lai et al., 2013; Lee et al., 2016). It has great potential for practical use due to its much higher resolution and acquisition speed, as well as being a noncontact and noninvasive measurement. The limitation of this method is that it cannot be carried out for some subjects who have low sensitiveness to light. In addition, a sudden decrease in the luminance may induce the onset of acute angle closure, which is dangerous for PACG.

According to the operating principle of indentation gonioscopy, we assumed that having the peripheral cornea deformed by an air puff, as how an ocular response analyzer and Corvis ST work, may also lead to morphological changes of the ACA, which can be detected by AS-OCT. Inspired by these, an air puff-based dynamic anterior segment optical coherence tomography (DAS-OCT) system was built and validated for evaluating the presence of PAS. The air-puff jet is directed on the cornea and deforms it; meanwhile, the dynamic changes of the morphology of the ACA are recorded by DAS-OCT, and certain structural parameters are calculated as quantitative indicators of PAS. With its high resolution, wide scanning range, and non-contact approach, it may have a great potential for clinical application.

## Methods

### Experimental Setup

**FIGURE 1:**
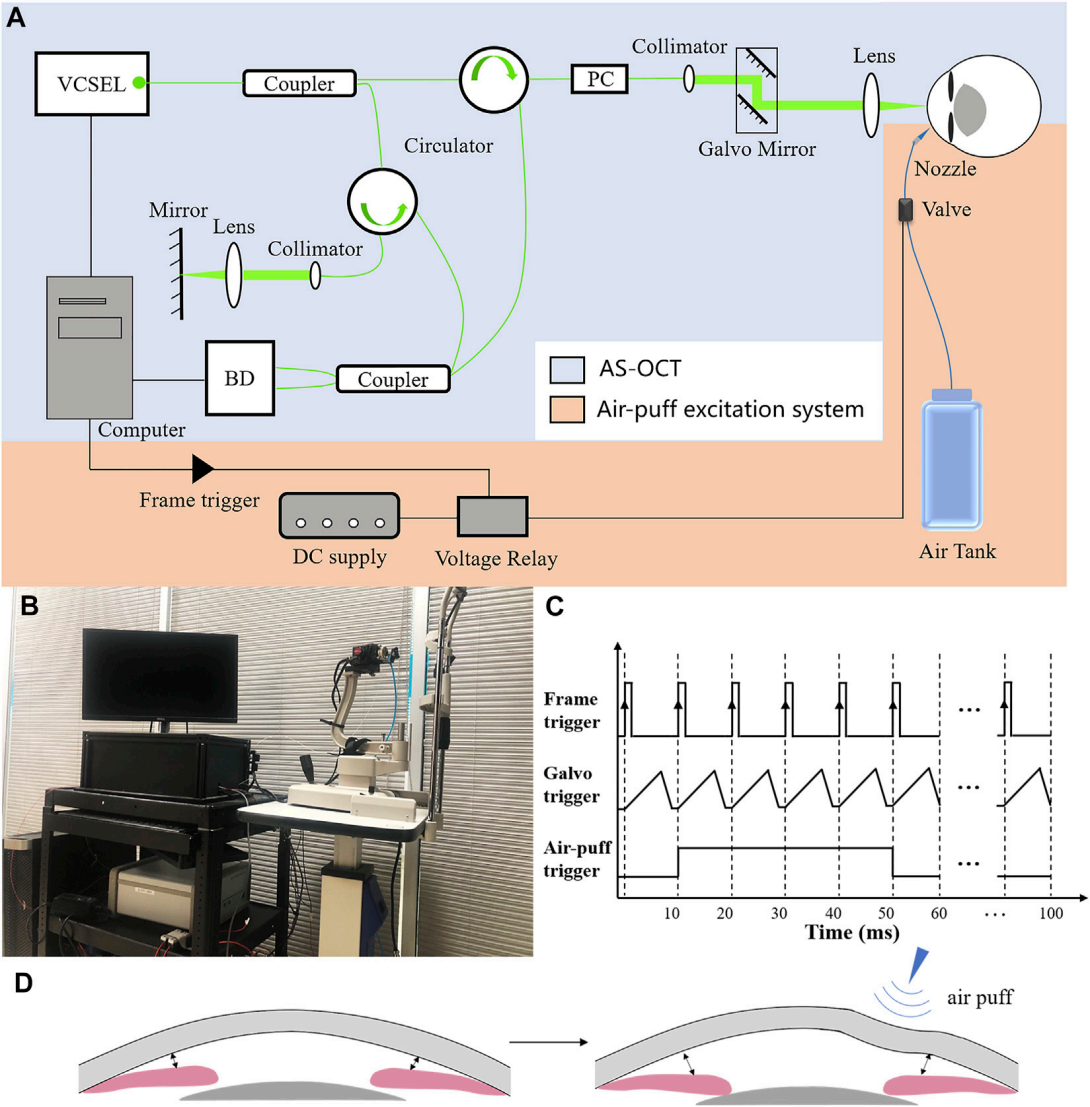
Principle diagram of the air-puff DAS-OCT system. **(A)**, Schematic representation of the DAS-OCT system. VCSEL, vertical cavity surface-emitting laser light source; PC, polarization controller; BD, balanced photodetector. **(B)**, Actual administration of the DAS-OCT system; **(C)**, Timing diagram of the DAS-OCT system; **(D)**, Schematic illustration of normal anterior segment structural changes caused by the air puff.

The custom-made air-puff DAS-OCT consisted of an AS-OCT and an air-puff excitation system (Figures 1A,B). The swept source OCT has been described in our previous studies (Dai et al., 2020; Dai et al., 2021), with an A-line scan rate of 200 kHz, a lateral scan range of 17.00 mm, an axial scan depth of 5.86 mm, and an axial resolution of 5.7 μm.

The air-puff excitation system included a DC power supply, a voltage relay, an air-dispensing valve, a nozzle, and an air tank. The high-speed solenoid valve with a response time in the millisecond level (35A-ACA-DDFA-1BA, Wixom, MI, United States) was controlled by the voltage relay and synchronized with the scanning of OCT through a frame trigger from a data acquisition card in the computer (Figure 1). In order to simulate the operating principle of indentation gonioscopy, a short duration (40 ms) medical grade air was delivered through a 0.85-mm inner diameter nozzle, which was aligned to normally point to the peripheral area of the cornea with a distance of 15 mm. The nozzle tip was inserted into a hole in a plate and positioned behind the front surface of the plate to prevent contacting with the cornea. The force of the air puff on the surface of the cornea was measured by a custom-built pressure tester based on a strain-sensitive film (Omega Engineering Inc., Norwalk, CT, United States). After calibration, the diameter of the application area of the force on the cornea is 8 mm, and the pressure is 23 kpa, which is less than the force in Corvis ST and ORA. The deformed peripheral cornea caused by the air puff transferred the pressure to the ACA through the aqueous humor, leading to a larger ACA width in case of normal subjects (Figures 1C,D).

### Data Acquisition and Processing

In this study, the structural images of the anterior chamber were acquired through a B-M scanning protocol. Each B-scan included 2048 A-lines forming a horizontal cross section of the anterior segment. A total of 10 B-scans were captured consecutively at the same scanning position, while the air-puff jet at the beginning of the 2nd imaging with a duration of 40 ms, in order to record the dynamic deformation of the anterior segment during each measurement.

**FIGURE 2:**
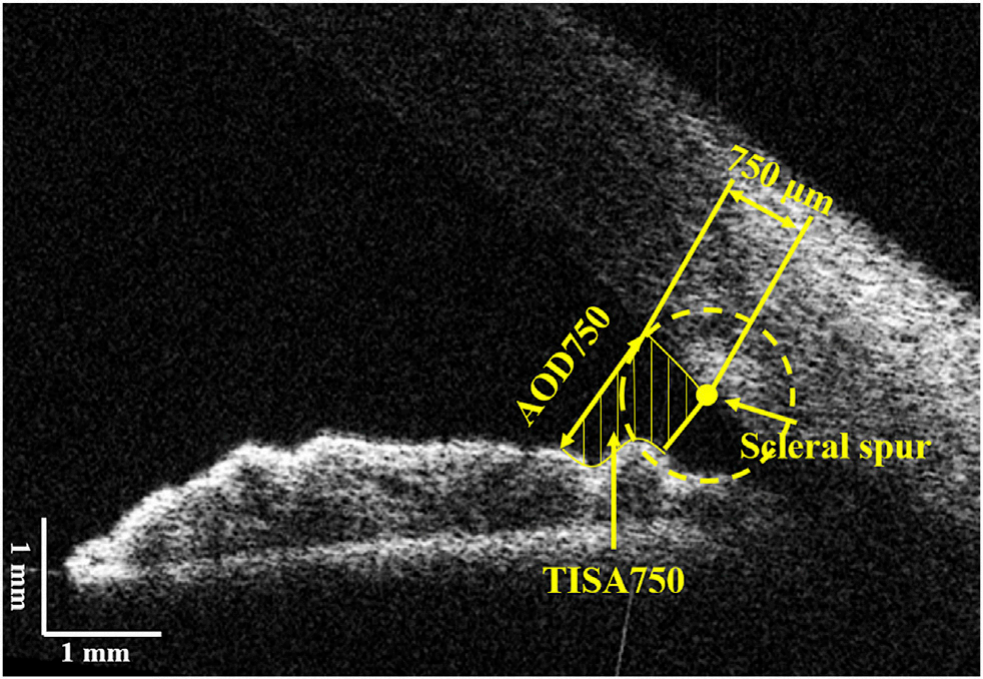
Illustration of the anterior chamber parameters. AOD750, angle opening distance at 750 μm from the scleral spur; TISA750, trabecular-iris space area at 750 μm from the scleral spur.

Structural ACA parameters were extracted from each OCT image using a custom-written algorithm described in our previous studies (Zhu et al., 2014; Dai et al., 2020; Dai et al., 2021). The scleral spur on both sides were manually marked after the image was corrected. The anterior and posterior surfaces of the cornea and the anterior surfaces of the iris and lens were automatically segmented by the algorithm. As shown in Figure 2, two ACA structural parameters were calculated, that are the angle opening distance at 750 μm to the scleral spur (AOD750) and the trabecular-iris space area at 750 μm anterior to the scleral spur (TISA750) (Dai et al., 2020; Dai et al., 2021). The parameter extracts from the image, in which the deformation of the cornea reach the maximum during the 10 consecutive OCT images, were selected as the values after the air puff, while the first image was considered as the base line. Then, the differences of each parameter between OCT images before and after the air puff were defined as the following, respectively:

$$\text{AOD}750_{\text{dif}}=\text{AOD}750_{\text{after}}-\text{AOD}750_{\text{before}},$$

$$\text{TISA}750_{\text{dif}}=\text{TISA}750_{\text{after}}-\text{TISA}750_{\text{before}}.$$

### Subjects and Grouping

Ten eyes from ten normal subjects (two females and eight males, aged 25.2 ± 0.78 years) and five eyes from five patients with ACG were selected from the Optometry School of Wenzhou Medical University. The right eyes of the volunteers were enrolled. Normal subjects, with best corrected visual acuity ≥1.0 and intraocular pressure (IOP) between 10 and 21 mmHg, were included and assigned into the normal group. Those with remarkable general or ocular diseases, history of eye surgery, poor fixation, and blepharophimosis were excluded. For the patients, gonioscopy was performed by a single experienced ophthalmologist. The eyes with static gonioscopy findings of a grade IV circumferential Scheie gonioscopy classification system in the horizontal direction and without ocular diseases that blocked the OCT scan beam were included. The ACA from patients were further assigned into the NPAS group and PAS group, according to their indentation gonioscopy results.

All volunteers underwent standard ophthalmic examinations including an examination of visual acuity, non-contact tonometer, and slit-lamp biomicroscopy. Then, they were asked to take the DAS-OCT examination. The ACA structural parameters on the same side and on the opposite side of the air-puff position were taken into analysis. All procedures were followed according to the World Medical Association’s Declaration of Helsinki.

### Statistical Analysis

The intraclass correlation coefficient (ICC) and Bland–Altman plots were used to characterize the repeatability of the measurements. For normal subjects, a paired *t* test was performed to compare the significant differences of ACA structural parameters before and after the air puff. For patients with ACG, independent-sample *t* test analysis was performed to compare the significant differences of ACA structural parameters between the NPAS group and PAS group.

## Results

### Measurement Repeatability

**FIGURE 3:**
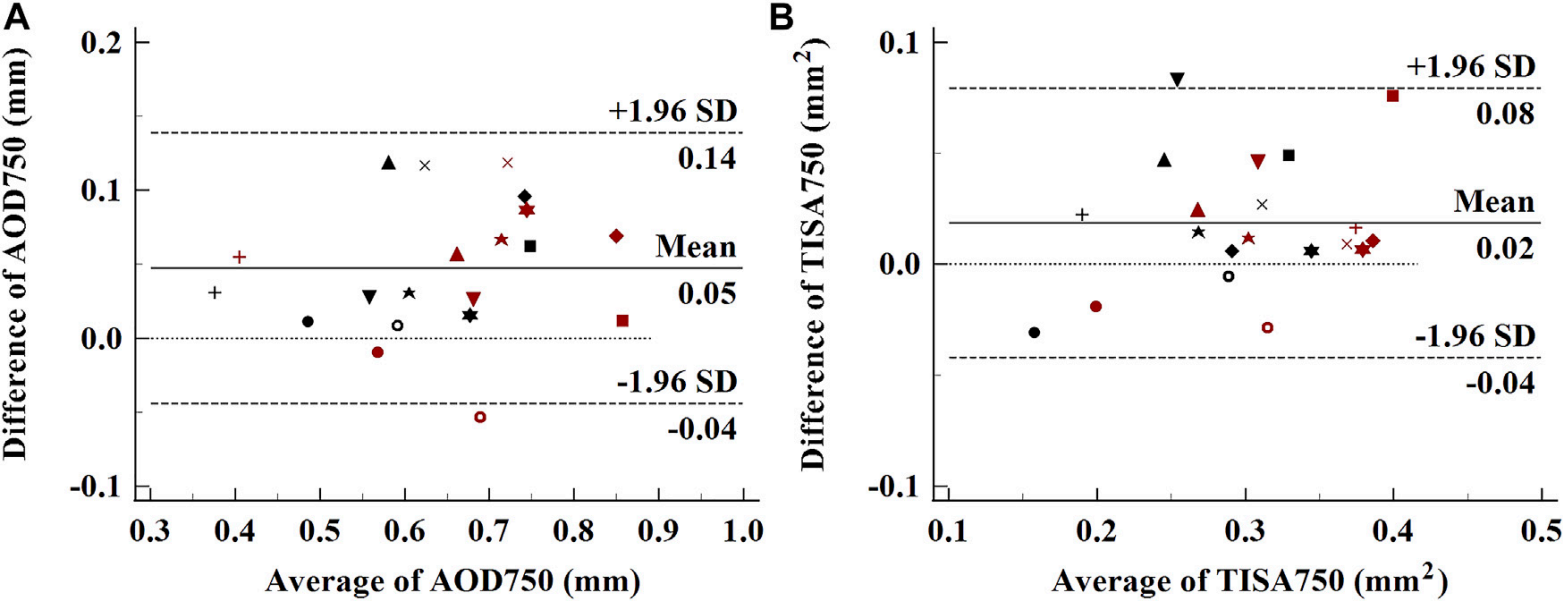
Bland–Altman plots of the repeatability of ACA parameters. **(A)**, AOD750; **(B)**, TISA750. Each subject was labeled by different shapes of spots. Black spots, parameters before the air puff; red spots, parameters after the air puff.

Each normal subject was measured twice under the same condition, and the obtained OCT images were processed by using one experienced operator. The same side of air puff excitation of ACA was analyzed, and the difference of structural parameters between before and after the air puff was calculated to verify the repeatability of the system. The Bland–Altman plots of AOD750 and TISA750 from the same side ACA (9’o clock) of each normal eye, including the data before and after the excitation, are shown in Figure 3. The ICCs of AOD750 and TISA750 are 0.881 and 0.877, respectively, exhibiting great repeatability.

### Availability of ACA Data

**FIGURE 4:**
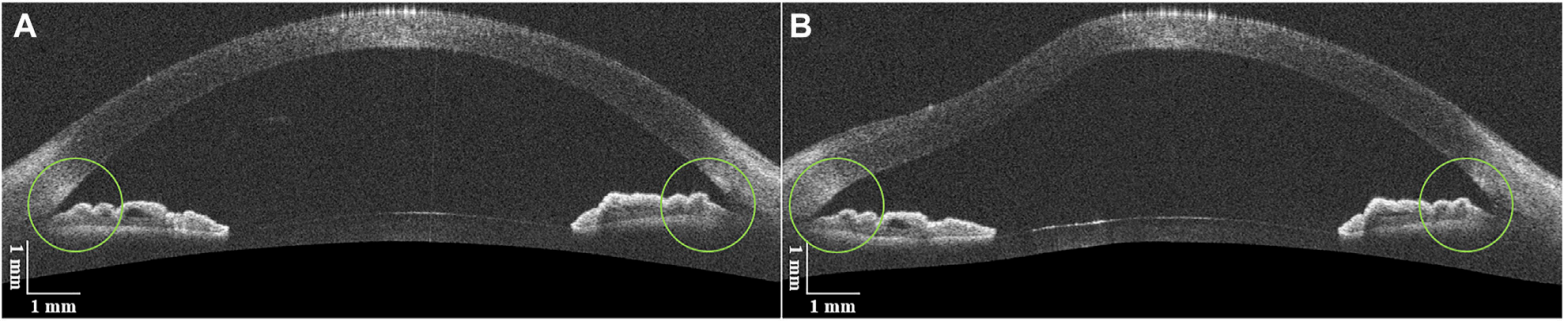
DAS-OCT images of a normal subject eye. **(A)**, image before the air puff; **(B)**, image after the air puff.

In order to verify the availability of the ACA on the same side of the air puff compared to the opposite side in one OCT image, ten normal subject eyes before and after the air puff were analyzed. As shown in Figure 4, the ACA on both sides opened wider after the air puff. For each side of the ACA, the average of the two measurements of AOD750 and TISA750 was used as an independent sample for analysis. The detailed AOD750 and TISA750 values for both sides of the ACA in all normal subject eyes are listed in Table 1. The two parameters both significantly increased after the air puff, indicating both sides of the ACA detected simultaneously by DAS-OCT can be used for the identification of PAS. Therefore, the data from both sides of the ACA in one OCT image will be equally included in this study.

**TABLE 1:** Structural parameters on both sides of the ACA in normal subjects.

	The same side (*n* = 10) mean ± SD	*p* Value	The opposite side (*n* = 10) mean ± SD	*p* Value
AOD750_before_/mm	0.599 ± 0.113	0.000*	0.575 ± 0.086	0.000*
AOD750_after_/mm	0.689 ± 0.131		0.686 ± 0.080	
TISA750_before_/mm^2^	0.268 ± 0.059	0.003*	0.264 ± 0.042	0.000*
TISA750_after_/mm^2^	0.330 ± 0.063		0.327 ± 0.050	

*p < 0.05, paired *t* test.

### Identification of PAS in Eyes With Angle Closure

**FIGURE 5:**
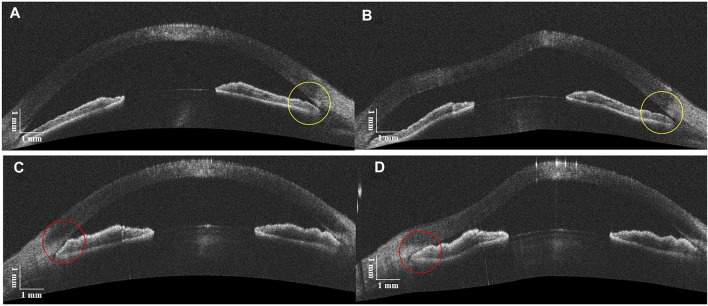
DAS-OCT images before and after the air puff. **(A)**, ACA with NPAS before the air puff; **(B)**, ACA with NPAS after the air puff (yellow circles). **(C)**, ACA with PAS before the air puff; **(D)**, ACA with PAS after the air puff (red circles).

The clinical characteristics of five patients with ACG are listed in Table 2, from which there are seven ACAs with NPAS and three ACAs with PAS. Figure 5 shows examples of DAS-OCT images of ACG patients. It is difficult to distinguish the chamber angle without PAS from that with PAS in an OCT image before the air puff, as shown in Figures 5A,C. Conversely, after the air puff, the NPAS ACA showed a very modest opening (Figure 5B), while the PAS ACA remained close (Figure 5D).

**TABLE 2:** Clinical characteristics of patients with angle closure glaucoma.

Subject	Gender	Age/year	Eye	IOP/mmHg	Indentation gonioscopy results	
					3’o clock position	9’o clock position
1	Male	52	OD	13.7	NPAS	NPAS
2	Female	67	OD	26.1	NPAS	PAS
3	Male	46	OD	17.2	NPAS	NPAS
4	Female	62	OD	22.4	PAS	NPAS
5	Male	35	OD	27.9	NPAS	PAS

**FIGURE 6:**
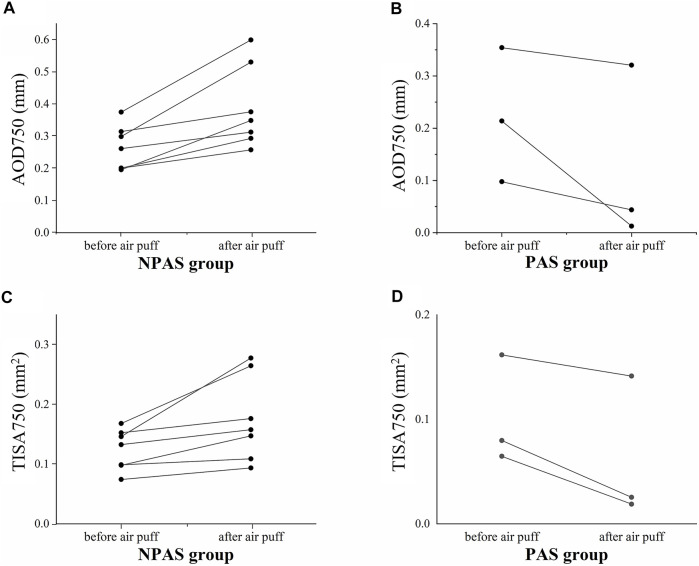
Variations of structural parameters of 10 closed ACAs (five eyes) before and after the air puff. **(A)**, AOD750 in the NPAS group; **(B)**, AOD750 in the PAS group; **(C)**, TISA750 in the NPAS group; **(D)**, TISA750 in the PAS group.

For patients with ACG, the variations of the two parameters before and after the air puff for individual ACA are shown in Figure 6. Increasing trends could be found in the NPAS group; on the contrary, the values were decreased in the PAS group. The detailed data were analyzed and are listed in Table 3. The independent-sample *t* test showed there were no significant differences of AOD750 and TISA750 before the air puff between the NPAS group and PAS group (P_AOD750before_ = 0.507 and P_TISA750before_ = 0.437). After the air puff, both AOD750 and TISA750 showed significant differences between the two groups (P_AOD750after_ = 0.026 and P_TISA750after_ = 0.049). Compared with the NPAS group, the calculated AOD750_dif_ and TISA750_dif_ were both smaller in the PAS group (P_AOD750dif_ = 0.005 and P_TISA750dif_ = 0.012).

**TABLE 3:** Comparisons of structural parameters between the NPAS and PAS groups before and after the air puff.

	NPAS (*n* = 7)	PAS (*n* = 3)	*p* Value
	Mean ± SD	Mean ± SD	
AOD750_before_/mm	0.264 ± 0.692	0.222 ± 0.128	0.507
AOD750_after_/mm	0.388 ± 0.128	0.126 ± 0.170	0.026*
AOD750_dif_/mm	0.124 ± 0.079	−0.096 ± 0.091	0.005*
TISA750_before_/mm^2^	0.124 ± 0.034	0.102 ± 0.052	0.437
TISA750_after_/mm^2^	0.175 ± 0.071	0.062 ± 0.067	0.049*
TISA750_dif_/mm^2^	0.051 ± 0.050	−0.040 ± 0.018	0.012*

*p < 0.05, independent-sample *t* test.

## Discussion

In this study, we described a DAS-OCT system to identify the presence of PAS in ACG patients by recording the dynamic process of morphological changes of the ACA excited by the air puff. It has been confirmed that AS-OCT is more sensitive and convenient in detecting angle closure than gonioscopy (Nolan et al., 2007; Sakata et al., 2008). But traditional AS-OCT cannot be an alternative to indentation gonioscopy, which requires a force applied to promote the ACA opening. In previous studies of our group, a multiple ACA-grade model based on AS-OCT images was demonstrated (Dai et al., 2020). Furthermore, we have reported an AS-OCT–based method for identifying the presence of PAS and evaluating the extent of PAS in a single eye based on the pupillary light reflex (Dai et al., 2021). Changes of ACA structural parameters such as the angle opening distance (AOD), trabecular-iris space area (TISA), and angle opening distance at the scleral spur (AODSS) have been analyzed. Nevertheless, the mechanism of ACA morphological changes caused by the brightness change was different from how gonioscopy works, so the application is limited because of the individual different reaction to light, which resulted in the inconsistent force from the iris contraction. The examination technology by the air puff is widely used in ophthalmology (Terai et al., 2012; Qin et al., 2019; Salouti et al., 2020), non-contact tonometer, and corneal biomechanical measuring instrument for instance and has been proved to be safe enough for eyes. The operating principle of the method proposed in this study was consistent with that of indentation gonioscopy, and more importantly, it provided objective and quantitative ACA parameters without much requirement for clinicians’ experience. This technology will reduce the training processing time; thus, it was more valuable in clinical application than gonioscopy.

The repeatability and feasibility of the technique were investigated in this article, showing the well reliability of our method. The fact that the ACA parameters on the same side of the air-puff position were also increased in normal subjects, as same changes as the opposite side, demonstrated that both sides of the ACA imaged during a single B-scan can be used. Hence, the measurement efficiency is improved compared with gonioscopy, which only evaluates the opposite side of the mirror. As shown in Figures 6B,D, the structural parameters decreased rather than increasing after the air puff for PAS, no matter on which side relative to the air-puff position. It may be caused by the backward deformation of the entire cornea under the force from the air puff, resulting in more adhesion between the cornea and iris. As shown in Table 3, there were no significant differences in AOD750_before_ and TISA750_before_ between the NPAS group and PAS group (P_AOD750before_ = 0.507 and P_TISA750before_ = 0.437), indicating it was difficult to distinguish PAS from appositional angle closure by static OCT images. The calculated AOD750_dif_ and TISA750_dif_ showed greater significances between two groups than AOD750_after_ and TISA750_after_ (P_AOD750dif_ = 0.005, P_TISA750dif_ = 0.012, P_AOD750after_ = 0.026, and P_TISA750after_ = 0.049). In addition, AOD750_dif_ showed a greater difference than TISA750_dif_. Based on the definition of TISA750, the value of that was strongly dependent on the accuracy of surface segmentation of the iris and corneosclera. On the contrary, AOD750 is simply defined as a distance between two points, which may have small detecting deviations compared with TISA750. Therefore, AOD750 _dif_ was found to be more sensitive for distinguishing PAS from NPAS.

For the current system, the imaging position was limited only in the horizontal direction of the anterior segment, that is, only 3’o clock and 9’o clock of the ACA can be assessed rather than of the whole 360°. Future studies will update the system to reach a faster acquisition speed and to acquire radial OCT images when the air puff can be applied on the corneal center. Thus, a map of the ACA structure can be formed, and the PAS in all positions can be identified, which would be more valuable in the clinic. Also, we are planning to expand the sample size in following studies so that a binary classification model could be established to evaluate the extent of PAS in patients with ACG.

In summary, we proposed an air-puff–based DAS-OCT system to identify the presence of PAS in a non-contact way. By the corneal deformation under the force of air puff, the ACA without PAS would open, while that with PAS remained close. The structural parameters including AOD750, TISA750, the difference of AOD750, and difference of TISA750 have been demonstrated as effective indicators. An objective parameter, the difference of AOD750, was found to be sensitive enough to distinguish non-PAS from PAS. The system described in this study was demonstrated to be effective for the diagnosis of PAS with good reliability and feasibility and will have potential application in the clinic.

## Data Availability

The raw data supporting the conclusions of this article will be made available by the authors, without undue reservation.

## Abbreviations

PACG, primary angle closure glaucoma; PAS, peripheral anterior synechia; NPAS, non-PAS; ACA, anterior chamber angle; AS-OCT, anterior segment optical coherence tomography; DAS-OCT, dynamic anterior segment optical coherence tomography; ACG, angle closure glaucoma; AOD750, angle opening distance at 750 μm to the scleral spur; TISA750, trabecular-iris space area at 750 μm anterior to the scleral spur.
